# Decision-Making under Uncertainty: How Easterners and Westerners Think Differently

**DOI:** 10.3390/bs12040092

**Published:** 2022-03-25

**Authors:** Wei Guo, Xin-Rong Chen, Hu-Chen Liu

**Affiliations:** 1 School of Management, Shanghai University, Shanghai 200444, China; guowei_shu@126.com; 2 Academy for Engineering and Technology, Fudan University, Shanghai 200433, China; chenxinrong@fudan.edu.cn; 3 School of Economics and Management, Tongji University, Shanghai 200092, China

**Keywords:** decision-making, uncertainty, preferences, ambiguity aversion, risk aversion, culture

## Abstract

It has long been known that Easterners exhibit more conservative attitudes, cautiousness behaviors, and self-control ability than Westerners; people in Eastern countries show stronger defensive reactions to societal threats than Western people. Are East Asians really risk averters or do some richer underlying preferences drive their behaviors in their decision-making under uncertainty? To answer this question, we examined the risk and ambiguity attitudes of East Asian populations in both gain and loss domains using classical behavioral economic experimental methods. Based on our sample of university students, we found that Easterners are more risk intolerant but more willing to accept ambiguous conditions than their Westerner counterparts in the gain domain. Perhaps surprisingly, Eastern people and Western people have a similar attitude toward risk and ambiguity in the loss domain. The higher level of risk aversion observed among East Asians may be due to the cultural difference between Western countries and Eastern countries. Historically, such risk aversion may make sense, because it would minimize the influence of numerous ecological and historical threats and socio-political practices. Our findings suggest that models that were designed to analyze and predict aggregate behaviors and markets may be ineffective for Eastern populations, and, in the future, it is of significance to develop appropriate representative agent models from the eastern perspective.

## 1. Introduction

Many important decisions are routinely made under uncertainty, covering different levels of society from single individuals and households to global organizations and countries [1,2]. East Asians engage in more discreet, conservative, and unadventurous behaviors than people from the European and American countries [3]. They have a lower rate of sexually transmitted diseases [4], a lower level of risky driving behavior [5], and lower transport mobility than their American counterparts [6]. Asian households have a lower credit delinquency rate compared to other racial/ethnic groups in the United States [7], and the Asian-Pacific region maintains a high average rate of gross domestic saving [8]. Also, Asian countries approach global governance largely in terms of self-help [9]. The COVID-19 mortality and morbidity rates of East Asians are much lower than those of Western Europeans and North Americans [10], a decrease that can be attributed to the higher rate of “risk aversion behaviors”. 

What are the psychological features causing the risk aversion behaviors of Asian people? Why do East Asians make choices that are clearly distinct from those of Euro-Americans? What feature of their decision-making leads to conservative attitudes and behaviors? To date, we have very few insights as to what accounts for the different behaviors between the East and the West. To understand behaviors in different cultures, previous research has revealed cultural variation in cognition [11], explored choice, and dissonance in different cultural contexts [12], and has demonstrated cultural differences in motivation [13]. Some studies identified a wide discrepancy across tight and loose cultures at the country level [14,15,16]. Gelfand et al. [14], for example, illustrated tightness–looseness as one factor to explain the variance between cultures. They found that tight countries have strong social rules and little tolerance for deviant behavior, whereas loose countries have weak social norms and greater tolerance for deviant behavior. In parallel, some studies analyzed different decision-making behavior across countries based on the dimension of individualism–collectivism [10]. It was indicated that the individualism–collectivism axis affects a person’s decision-making not only in perceiving a problem but also in generating and selecting alternative strategies [17]. Individuals in the individualistic culture often pursue individual independence and achievement, while those in the collectivistic culture tend to hold views that are more easily affected by the surrounding environment [18]. 

According to Hofstede [19], culture is defined as ‘the collective programming of the mind that distinguishes the members of one group or category of people from another’. National culture is a set of values and beliefs that people within a society pass on relatively unchanged from one generation to the next [20]. Various frameworks have been developed to describe national cultures [21,22]. Among them, Hofstede’s model is the popular one, which characterizes national culture using six dimensions, i.e., individualism, power distance, uncertainty avoidance, masculinity, long-term orientation, and indulgence vs. restraint [21]. In addition, national culture has been shown to affect economic and financial behaviors in different contexts. For example, Frijns et al. [20] investigated the effect of national culture on corporate risk-taking and found that individualistic cultures incentivize corporate risk-taking. Hoang et al. [23] reported that the COVID-19 pandemic’s negative impact on stock market returns is relieved in societies where people are more collectivistic and cooperative and less tolerant towards uncertainty. An empirical analysis in [24] showed that the national culture is an important country-specific factor affecting international portfolio choices.

Based on the above discussions, the driving research questions of this paper are as follows: How do Easterners and Westerners think differently in their decision-making under uncertainty? Why do East Asian people exhibit more conservative attitudes and behaviors in uncertain decision-making? To understand these questions, it is important to investigate the decision-making process as a function of culture. Understanding the impact of culture on risk preferences is an important step in forecasting the decisions made by East Asians in political and economic processes at both global and local levels. A growing body of psychological and economic evidence indicates that people exhibit risk and ambiguity aversion in uncertain decision-making; however, previous studies have normally investigated the risk-related behaviors from the western perspective. In this article, we employed a well-validated choice task grounded in behavioral economic theory [25,26,27] to explore cultural differences in uncertain decision-making based on a sample of Easterners.

The main contributions of the current study are that we investigated whether Easterners and Westerners are characterized by different attitudes towards risk and ambiguity, and further examined the influential elements of cultural context on decision-making under uncertainty. In the following section, the methodological approach is presented, including a description of the applied research design using standard experimental economic methods. Thereafter, the results of the experiment are summarized and discussed. Finally, concluding remarks of this study are provided and recommendations for future research are proposed.

## 2. Materials and Methods

### 2.1. Participants

A total of 120 healthy right-handed subjects between 22 and 32 years of age participated in the experiment: 59 East Asians (20 female) and 58 Euro-Americans (20 female). The sample size was determined from past work using the same task together with physiological recordings [27,28,29]. The subjects were recruited in Shanghai University and Tongji University, via social media platforms among graduate students. We balanced the number of men and women in each subject group and at each location. All participants signed an informed consent. 

All potential participants were screened for psychotropic medications known to influence decision-making under risk, such as medications for attention deficit disorder, psychiatric disorders, and cognitive/neurological disorders. Any possible participant who had recently been medicated for one or more of these conditions was excluded from participating in the experiment. 

The main reason for the choice of sample based on the above screen criteria is that the influence of factors on the experiment results such as neurological illnesses, handedness, age, and medication can be avoided.

### 2.2. Main Task

On arrival at the testing site, participants were seated individually in front of private computer stations where they received extensive instructions and practice trials. After they understood the task, a comprehension quiz regarding the stimuli and payment rules was given for each participant. Participants were allowed to proceed to the experiment only when they correctly answered all comprehension questions and felt comfortable with the task.

**Figure 1:**
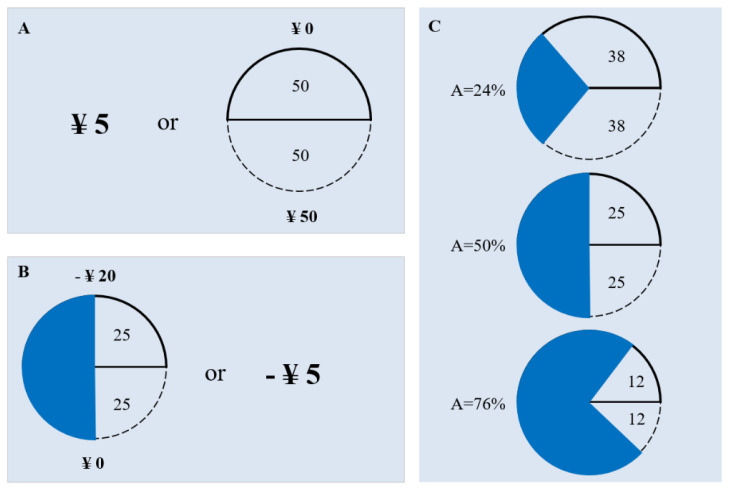
Experimental design. (**A**) Example of risky gain trial. The participant chose between RMB 5 and a lottery of winning RMB 50 or nothing with an equal probability. (**B**) Example of ambiguous loss trial. The participant has a choice between losing RMB 5 and a lottery of losing RMB 20 with unknown probability. (**C**) Ambiguity levels used in the experiment.

The experiment consisted of two sessions. In the first session, we evaluated subjects’ risk and ambiguity attitudes using a standard incentive-compatible technique commonly used in the economics and neuroscience literature [26,30]. This session included 120 sequential trials that presented a choice between pairs of two monetary options. Among them, 60 were between certain and uncertain gains, and 60 were between certain and uncertain losses. For the gain trials, one option was a certain payoff of RMB 5, the other was a lottery with a probability to pay more than RMB 5 and a probability to pay RMB 0. For the loss trials, one option is a certain to lose RMB 5, the other is a lottery with a probability to lose more than RMB 5 and a probability to lose RMB 0. Moreover, the outcome probability was known (risk; Figure 1A) or partially unknown (Figure 1B,C) in the gain and loss trials. Across trials, the details of the lottery were varied systematically to determine how the outcome probability (25%, 50%, and 75%), the monetary amount (RMB ± 5, RMB ± 8, RMB ± 20, RMB ± 50, and RMB ± 125), and the ambiguity level (24%, 50%, and 74% ambiguity around a probability of 50%) influenced choices. Probability and ambiguity levels are fully crossed with the gain and loss amounts, yielding 60 unique lotteries. Each unique selection was presented two times, counterbalancing the side on which the lottery option appeared on the computer screen, giving a total of 120 selections per subject. In each trial, participants indicated their choice via pressing a corresponding mouse button. Four blocks of 30 trials were presented interleaved with symmetrically structured blocks of gain and loss trials. Moreover, the sequence of gain and loss blocks was counterbalanced within each subject group. 

Every lottery was either risky or ambiguous, allowing us to assess each subject’s aversion to known and unknown monetary risks. Figure 1A depicts a risky trial. Here, a subject chooses between a certain RMB 5 and a lottery with a 50% chance of winning RMB 50 or RMB 0. Subjects were told that each image depicted a bag of 100 balls labelled as A and B, the number of balls of each type denoted by the size of corresponding area and the number marked in it. In our experiment, we use balls labelled A and B, rather than following the traditional practice of using red and blue balls to avoid the bias of color preference [31].

Figure 1B depicts an ambiguous trial, a choice between a certain RMB 5 or a lottery losing RMB 0 or RMB 20 with an ambiguous probability. To create ambiguity, a blue occluder covering 50 of the balls is displayed; the subject knows that there are at least 25 A and 25 B balls. The remaining 50 can be any combination of A and B, implying that the odds of losing RMB 0 can be anywhere from 25% to 75%. Figure 1C presents the three possible ambiguous lotteries. Increasing cover size reduces the information about outcome probability, thus raising the level of ambiguity.

In the second session, participants completed a detailed demographic questionnaire and were assessed by a series of physical assessments [Behavioral approach scores (BAS)/Behavior inhibition scores (BIS) scales [32], the Barratt Impulsiveness Scale (B11) [33], and the Domain-Specific Risk-Taking Scale for adults [34]. All measures were taken after participants had completed the main task. 

### 2.3. Payment and Estimation

As previous experiments indicated that subjects may behave differently when their choices are hypothetical [17], we made the decisions consequential. After making all the 120 pairwise decisions, a participant would be paid based on the total amount of money they earned at a fixed rate of one Yuan per 10 points. Each participant was endowed with RMB 125 at the beginning of the experiment. In addition, participants received a flat fee RMB 40 for participating in the questionnaire session. 

Participants who selected a lottery (a possible win of RMB 5) over a certain win of RMB 5 or a certain loss of RMB 5 over a lottery (a possible loss of RMB 5), more than 50% of the time were excluded from further analysis because we could not in principle infer their risk and ambiguity preferences [26,35]. There were three such participants, one Asian subject and two Euro-American subjects.

## 3. Results

**Figure 2:**
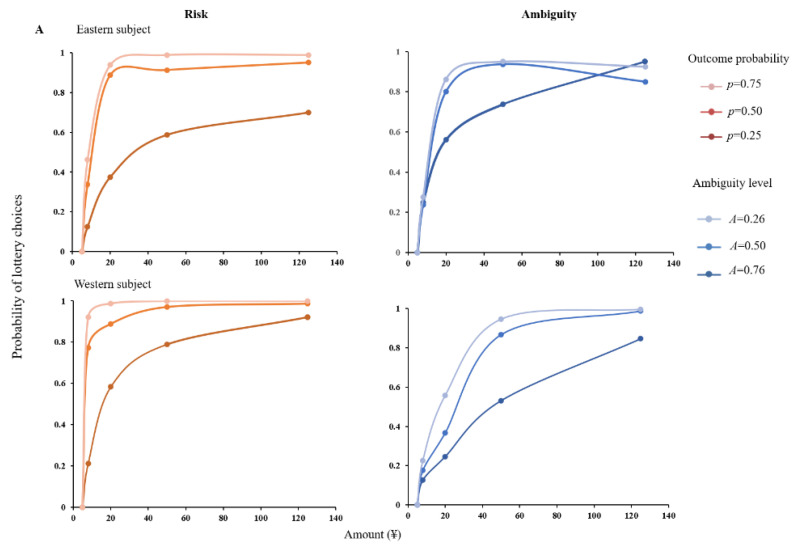

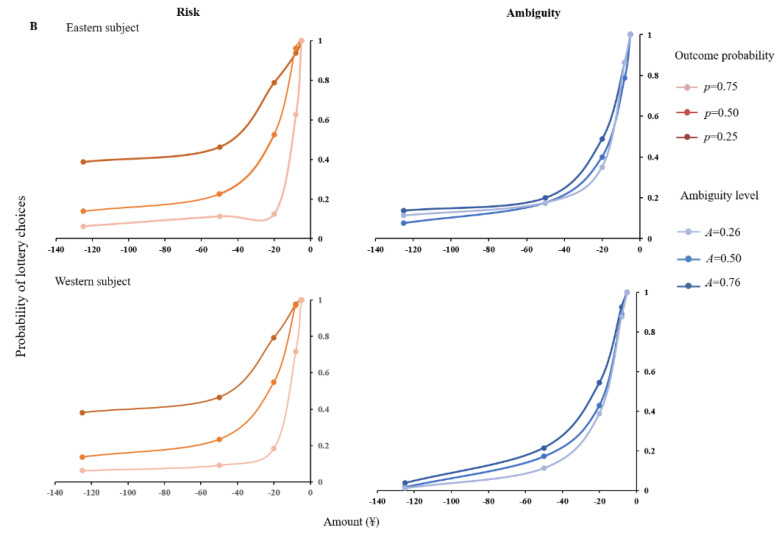
Subjects’ choice behaviors. The graphs represent the proportion of trials in which a subject chose the lottery over the certain amount, i.e., RMB 5 (**A**) or RMB −5 (**B**), as a function of the given amount in risky (**Left**) and ambiguous (**Right**) tests. Points denote actual choice behaviors. Smooth lines are a result of fitting the choice data to the subjective theoretical model.

To quantify uncertainty attitudes of the two subject groups, we calculate the proportion of times each chose a lottery over the certain option for every risk level. Figure 2 shows the proportion of trials in which subjects accepted the lottery as a function of lottery amount in risky and ambiguous trials. In the gain domain, both the Easterners and the Westerners displayed risk aversion by choosing the certain amount over lotteries with higher average payoff, as shown in Figure 2A. However, these subjects differed in the level of their risk aversion; East Asians are more risk-averse than their Euro-American counterparts. Though the Westerners always preferred the chance of RMB 125 over the certain RMB 5, the Easterners did not. When the outcome probability is low (i.e., 0.25), East Asians are more risk-averse than Euro-Americans. In addition, both the Easterners and the Westerners displayed some ambiguous aversion in the gain domain. However, they vary a lot in their attitudes towards ambiguity. The western subjects made significantly different choices under the three ambiguity levels. The eastern subjects, however, showed a weak effect of ambiguity. Their choices of lotteries at 50% ambiguity were similar to their choices of risky lotteries at a 50% winning chance. Specifically, these subjects are risk-taking and preferred to choose a lottery over a certain option when the average payoff is high.

As Figure 2B shows, in the loss domain, both the Easterners and the Westerners displayed some risk appetite by choosing lotteries over the certain amount with bigger average loss. Moreover, they are similar in the level of their risk appetite. In addition, both the Easterners and the Westerners are ambiguity neutral and showed a weak effect of ambiguity. That is, these subjects made similar choices regardless of the levels of ambiguity and behaved as if the winning probability is 50% in the ambiguous lotteries.

**Figure 3:**
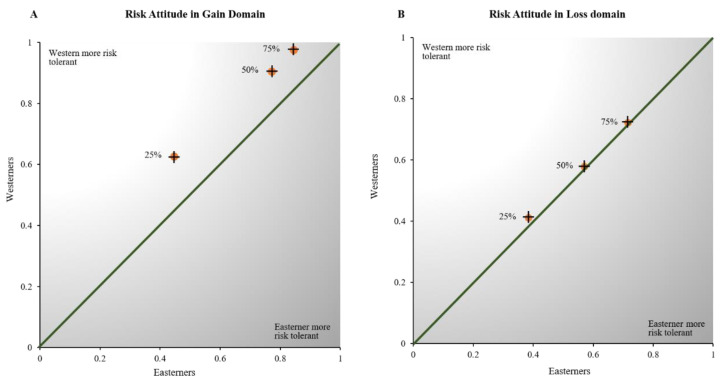
Comparison of risk attitudes in the Easterners and the Westerners. In (**A**) each point represents the average proportion of times that a risky lottery was selected over the certain amount RMB 5 by the Easterners and the Westerners. In (**B**), each point represents the average proportion of times that a risky lottery was selected over the certain amount RMB −5 by the Easterners and the Westerners.

Figure 3 shows the overall proportion of risky choices selected by the Easterners against the same measure in the Westerners at each of the three considered risk levels. If the Easterners are more risk intolerant than the Westerners, then these points should lie above the main diagonal of the graph. Figure 3A indicates that this is the case in the gain domain; all of the points lie above the diagonal line. The East Asians clearly accept less risky lotteries than do their Euro-American counterparts in the gain domain. In other words, the Westerners are more risk-seeking than the Easterners in the gain domain. A two-way ANOVA verifies that these overall culture-related risk differences in the gain domain are statistically significant (*p* = 0.002) in our sample. On the other hand, Figure 3B displays that both the Easterners and the Westerners have a similar attitude toward risk in the loss domain.

**Figure 4:**
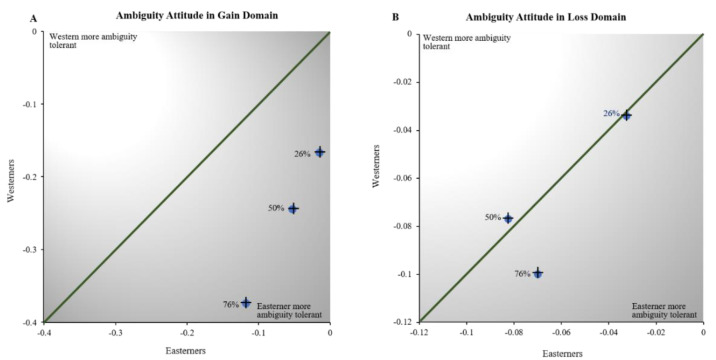
Comparison of ambiguity attitudes in Easterners and Westerners. In (**A**), each point plots the average ambiguity attitude of the Easterners against the ambiguity attitude of the Westerners at each level of ambiguity in the gain domain. In (**B**), each point plots the average ambiguity attitude of the Easterners against the ambiguity attitude of the Westerners at each level of ambiguity in the loss domain.

To assess ambiguity attitudes of our subject populations, the risk attitude of each individual should be considered since the ambiguous lotteries are a combination of risk (at 50%) and ambiguity [27]. Hence, we computed the difference between the proportion of times that a lottery of each ambiguity level was selected and the proportion of times that risky lotteries with a 50% chance of winning were selected. Figure 4 shows the risk-corrected overall proportion of ambiguous choices made by the Easterners against the same measure in the Westerners at each of the three ambiguity levels we examined. If the Easterners are more ambiguity intolerant than the Westerners, these points should lie above the main diagonal of the graph. As shown in Figure 3A, this is not the case in the gain domain. All the data points lie below the diagonal line. That is, East Asians are more likely to accept ambiguous choices than their Euro-American counterparts. A two-way ANOVA confirms that these overall culture-related differences in ambiguity attitudes are statistically significant (*p* = 0.001) in the gain domain. However, Figure 3B indicates that, in the loss domain, the ambiguity attitudes of the two subject groups are not significantly different at the lower ambiguity levels (A = 0.26 and A = 0.50). However, when the ambiguity level is very high (A = 0.76), the East Asians are more willing to accept ambiguous lotteries than their Euro-American counterparts in the loss domain. This culture-related difference of ambiguity attitude in the loss domain is statistically significant (*p* = 0.011) according to a two-way ANOVA.

To measure the risk and ambiguity attitudes of every subject, we model the expected utility (*U*) of every option by extending the classical power utility function considering the ambiguity effect. This function was first proposed by Gilboa and Schmeidler [36] and frequently employed in many previous studies [25,27].

(1) $$U\left(p,A,x\right)=\{\begin{array}{c} \left(p-\beta \times \frac{A}{2}\right)\times x^{\left(\alpha +1\right)},\;\;\;\mathrm{if}\;x\geq 0\\ 1-\left|p-\gamma \times \beta \right|\times {\left(-x\right)}^{\left(\alpha +1\right)},\;\;\;\mathrm{if}\;x>0 \end{array}$$

where *x* is the lottery outcome, *p* is the objective winning or losing chance, *A* is the ambiguity level, *α* is an individual risk attitude parameter, *β* is an individual ambiguity attitude parameter, and *γ* is the risk aversion coefficient of a decision maker. A subject with *α* = 0 would be risk neutral. In gain trials, a risk seeking subject would show an *α* > 0, and a risk averse subject would show an *α* < 0. In loss trials, *α* < 0 indicates risk seeking, whereas *α* > 0 indicates risk aversion. An ambiguity neutral subject will have a *β* = 0. An ambiguity averse (pessimistic) subject would show a *β* > 0 in the gain trials and *β* < 0 in the loss trials; an ambiguity-seeking (optimistic) subject would show a *β* < 0 in the gain trials and *β* > 0 in the loss trials. 

Bedsides, we employ the following probabilistic choice function to fit the choice data for the two subject groups [27,37]:

(2) $$\mathrm{Pr}\left(chose\;risky\right)=\frac{g\left(U_{risky}\right)}{g\left(U_{risky}\right)+g\left(U_{safe}\right)}$$

where *g* is the gaussian filter function used to eliminate the outliers.

**Figure 5:**
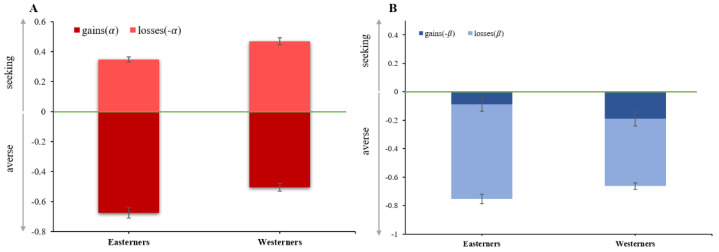
Estimated (**A**) risk and (**B**) ambiguity in the gain and loss domains. The green lines denote risk neutrality (*α* = 0) in *A* and ambiguity neutrality (*β* = 0) in *B*.

Figure 5 displays the group results of the two representative subjects in the gain and loss domains. Figure 5A shows the risk aversion degree (*α*) observed in the Easterners (*α* = −0.676 ± 0.08; mean ± SE) and the Westerners (*α* = −0.505 ± 0.07), with *α* = 0 indicating risk neutrality. In the gain domain, both the eastern and western subjective groups are risk averse, but the Easterners are more risk averse than the Westerners (Wald test, *p* = 0.0214). A similar pattern is observed in the loss domain, which shows the risk aversion degree (*α*) observed in Easterners (*α* = 0.347 ± 0.06) and Westerners (*α* = 0.468 ± 0.07). As in the nonparametric analysis, the Easterners are more risk averse than the Westerners (Wald test, *p* = 0.0116) in the loss domain. 

Figure 5B shows the degree of ambiguity aversion (*β*) observed in the Easterners and the Westerners. In the gain trials, the Easterners in this sample are more ambiguity-tolerant (*β* = 0.0863 ± 0.05) than the Westerners (*β* = 0.1881 ± 0.08; Wald test *p* = 0.0135). The Westerners exhibit more randomness degree in their behaviors (Wald test *p* = 0.0136) in the gain trials. In the loss trials, however, the Easterners were ambiguity-neutral and they tolerate less ambiguity (*β* = −0.6673 ± 0.04) than the Westerners (*β* = −0.4753 ± 0.06; Wald test *p* = 0.0206). 

The obtained results are robust to other model specifications after controlling the socioeconomic and demographic factors. In the following analysis, we show that the culture-based differences determined cannot be attributed to other demographic or psychological characteristics observed in our study.

First, we analyzed the psychological attraction to desired objects by each individual’s sensitivity to signals of reward and punishment, in terms of the BAS and BIS, as shown in Table 1. In our work, each individual’s BIS/BAS scores were controlled in the fitting process to investigate whether there was an independent effect of the Easterners and the Westerners on risk and ambiguity attitudes. When these covariates were added, we found that there was still a significantly greater tolerance for risk and ambiguity in the Westerners. 

We analyzed the attitudes to risk and ambiguity by controlling the individual differences in impulsivity and used B11 as independent covariates (Table 2). From the table, the systematic differences in impulsivity characteristics between the Easterners and the Westerners are not statistically significant. In a similar way, we found that the Easterners are not less risk-seeking and are significantly more ambiguity-tolerant than the Westerners when the differences in sex, total household wealth, and personal financial situation are considered (Table 3).

## 4. Discussion

In this study, we used a well-validated behavioral economic task to analyze the decision-making behavior of an Eastern population in the presence of uncertainty. The difference of risky actions across the culture is more complex than previously expected, showing conflicting patterns of culture-related changes in both risk aversion and ambiguity aversion. Based on our sample of university students, the results suggest that East Asians are more risk-averse than Euro-Americans in the gain domain, especially when the outcome probability is very low. However, interestingly, the Eastern subjects showed a weak effect of ambiguity, and the Western subjects have great differences in their choices under different ambiguity levels. We identified that the Easterners and the Westerners displayed similar risk appetites in the domain of losses. Moreover, it was found that both the Easterners and the Westerners are ambiguity-neutral and showed a weak effect of ambiguity. These results are robust to different data analytic methods and remain significant when we control for systematic differences in socioeconomic and psychological factors.

*Cultural differences.* Normally, research on risk attitudes in different countries has focused on differences in cultural norms, political ideology, values, religious beliefs, and problematic situations [20,24,38]. Since cultural tightness, i.e., the strength of social norms [39], can emerge at a societal level as a consequence of past experienced events [14], it is likely to determine human cognition and behavior, as well as neural activity in the face of uncertainty. Tight countries such as Singapore, Japan, and China have many strict social norms and are in general less accepting of deviant behavior, while loose countries such as the US, Italy, and Brazil can commonly be described as cultures with few social rules and a high tolerance for deviant behavior [10,14]. Tightness–looseness can affect not only many areas of life but also the development of personality traits. Prior research has demonstrated that tight cultures normally have stricter parent-child relationships, high-monitoring education strategies, and more media control [15,40]. In addition, people in tight societies are generally more conforming, have a higher need for stability, and prefer to avoid risks [39,41]. People from tight cultures are more defensive in the face of uncertainty as compared to people from loose cultures. Putting these insights together, it can be concluded that cultural tightness is a major factor underlying differences in people’s risk attitudes between East Asians and Euro-Americans. 

*Gains and Losses.* Previous psychological studies indicated that individual attitudes toward risk and ambiguity in the gain and loss domains are not correlated [42,43]. That is, they are two separate characteristics differentially associated with personality traits. The risk and ambiguity attitudes in the domain of gain are not directly applicable to the domain of loss [26,44]. Moreover, a rich body of evidence [2,45,46] has shown that risk and ambiguity attitudes have a greater sensitivity to losses than to gains. Compatible with previous findings, our study detected very different risk attitudes in gain and loss domains at the within-subject level. Therefore, individual uncertainty (risk and ambiguity) attitudes in the gain domain cannot be generalized to the loss domain both for the Easterners and the Westerners.

*Risk and Ambiguity Attitudes.* The previous evidence is mixed for the correlation between risk and ambiguity attitudes. On the one hand, some researchers found little or no association between risk and ambiguity attitudes. Curley et al. [47] and Levy et al. [25] did not obtain the evidence of a correlation between risk and ambiguity attitudes. Levy and Schiller [42] and Cohen et al. [48] indicated a weak correlation between risk and ambiguity attitudes across individuals. Di Mauro and Maffioletti [49] found a statistically significant correlation only for extreme probabilities. On the other hand, a positive correlation between risk and ambiguity attitudes was reported such as in the laboratory experiment [50,51] and the asset market context [52]. A strong correlation between ambiguity and non-expected utility risk attitudes was found in [53]. In addition, domain specific correlations between risk and ambiguity attitudes were identified in some studies. For instance, Lauriola and Levin [54] reported that the correlation was especially strong in the loss domain rather than in the gain domain. However, Chakravarty and Roy [55] found a positive relation in the gain domain but no relation in the loss domain, and Tymula et al. [26] indicated a weak link between risk and ambiguity attitudes in the gain domain. According to the results of [26,55], our findings showed that individual risk and ambiguity attitudes are correlated in the gain domain in Easterners; they are only weakly correlated in the loss domain in Eastern and Western people. 

*Policy Implications.* Understanding how East Asians and Euro-Americans are different in their risk and ambiguity attitudes is an issue of significant importance but one that has received limited attention. People anywhere are commonly assumed to have the right and the ability to make their own decisions to maximize their welfare. In previous studies, however, culture-related heterogeneity in risk and ambiguity attitudes has rarely been considered in modelling aggregate behavior and markets. In current behavior-predicting models, researchers have tended to use western subjects and build forecasts that overlook the differences between the east and the west in preferences—which we show here are very important. The data obtained in this study indicate that these westerner-based models may not be applicable to the Eastern population. The finding that risk and ambiguity attitudes in the loss domains do not differ much for the Easterners and the Westerners, however, is good news for most models. It suggests that the representative agent methods to market design, policy, and macro analysis may be appropriate for loss scenarios of our daily decision-making. However, in the gain domain, East Asians are clearly distinct from their Euro-American counterparts in our research, and this strongly suggests the importance of developing representative agent models based on the eastern subjects.

Our results suggest that the thinking between Eastern and Western populations is different in risk and ambiguity preferences when decision-making under uncertainty, implying that suitable policy interventions may be beneficial for individuals, organizations, and countries. For instance, a wide use of vaccines among the population is an effective way to control the spread of the COVID-19 pandemic. However, vaccines are normally rejected by a significant majority of Chinese people. A good way to improve COVID-19 vaccine acceptance is providing detailed explanations and instructions so that the risk and ambiguity level can be reduced.

## 5. Conclusions

In the present paper, the risk and ambiguity attitudes of East Asian and Euro-American participants in both gain and loss domains were investigated with a behavioral economic experimental method. A classical selection task commonly used in behavioral economics was utilized to analyze the cultural discrepancies in uncertain decision-making. Based on our sample of university students, we found that Easterners are more risk intolerant but more willing to accept ambiguous conditions than their Westerner counterparts in the gain domain. In the loss domain, however, Easterners and Westerners have a similar attitude toward risk and ambiguity. The higher level of risk aversion observed among East Asians may be a result of the cultural difference between Western and Eastern countries.

We close with some important suggestions for future work. First, considering that subject characteristics may influence inter-subject variation in uncertain decision-making, it would be preferable to have a wider database (more participants) in the future to have a more solid foundation for the behavioral findings. Second, this research revealed how Easterners and Westerners think differently in their decision-making under uncertainty. However, what cause the differences has not been explored in this study. Therefore, future studies will be required to understand why Easterners and Westerners make different choices and what neural mechanisms might be associated with this difference in risk preference.

## Figures and Tables

**Table 1:** Analysis of risk and ambiguity attitudes, including BIS and BAS scores as covariates.

	*α*	*β*
Easterners	−0.154 *	−0.463 **
	(−1.97)	(−4.22)
BAS drive	−0.012	−0.042
	(−0.41)	(−1.16)
BAS fun	−0.003	0.007
	(−0.18)	(0.25)
BAS reward	−0.057 *	0.154
	(−1.33)	(2.21)
BIS	0.021	−0.024
	(0.87)	(−0.63)
Constant	0.826	0.544
	(3.12)	(0.87)

Z statistics in parentheses. Robust SEs clustered on subject. * *p* < 0.05; ** *p* < 0.01.

**Table 2:** Analysis of risk and ambiguity attitudes, including B11 as covariates.

	*α*	*β*
Easterners	−0.298 **	−0.322 **
	(−3.36)	(−2.23)
First-order B11 factors		
Attention	0.022	−0.075
	(0.83)	(−1.13)
Motor	−0.014	0.043
	(−0.38)	(0.64)
Self-control	0.036	0.088
	(0.92)	(0.79)
Cognitive complexity	−0.058	0.052
	(−1.65)	(1.13)
Perseverance	−0.006	−0.032
	(−0.17)	(−0.32)
Cognitive instability	0.014	−0.018
	(0.42)	(−0.37)
Second-order B11 factors		
Attentional	0.035 *	−0.017
	(1.43)	(−0.16)
Motor	−0.118 *	0.015
	(−2.18)	(0.12)
Nonplanning	0.018	−0.065
	(1.14)	(−1.45)
Constant	1.056 *	0.794
	(3.42)	(1.18)

Z statistics in parentheses. Robust SEs clustered on subject. * *p* < 0.05; ** *p* < 0.01.

**Table 3:** Analysis of risk and ambiguity attitudes, including socioeconomic factors as covariates.

	*α*	*β*
Easterners	−0.149 *	−0.432 **
	(−1.76)	(−3.32)
Male	0.073	0.086
	(0.71)	(0.63)
Household wealth	0.000	0.000
	(0.12)	(−0.18)
Personal financial situation	−0.017	−0.002
	(−0.56)	(−0.03)
Constant	0.487	0.243
	(1.41)	(0.42)

Z statistics in parentheses. Robust SEs clustered on subject. * *p* < 0.05; ** *p* < 0.01.

## Data Availability

Not applicable.
